# Fast comparison of genomic and meta-genomic reads with alignment-free measures based on quality values

**DOI:** 10.1186/s12920-016-0193-6

**Published:** 2016-08-12

**Authors:** Matteo Comin, Michele Schimd

**Affiliations:** Department of Information Engineering, University of Padova, Via Gradenigo 6/A, Padova, Italy

**Keywords:** Meta-genomes, Phylogeny without assembly, Alignment-free measures, Reads quality values

## Abstract

**Background:**

Sequencing technologies are generating enormous amounts of read data, however assembly of genomes and metagenomes remain among the most challenging tasks. In this paper we study the comparison of genomes and metagenomes only based on read data, using word counts statistics called alignment-free thus not requiring reference genomes or assemblies.

Quality scores produced by sequencing platforms are fundamental for various analyses, moreover future-generation sequencing platforms, will produce longer reads but with error rate around 15 %. In this context it will be fundamental to exploit quality values information within the framework of alignment-free measures.

**Results:**

In this paper we present a family of alignment-free measures, called *d*^*q*^-type, that are based on *k*-mer counts and quality values. These statistics can be used to compare genomes and metagenomes based on their read sets. Results show that the evolutionary relationship of genomes can be reconstructed based on the direct comparison of theirs reads sets.

**Conclusion:**

The use of quality values on average improves the classification accuracy, and its contribution increases when the reads are more noisy. Also the comparison of metagenomic microbial communities can be performed efficiently. Similar metagenomes are quickly detected, just by processing their read data, without the need of costly alignments.

## Background

With the development of sequencing technologies, lots of reads can be easily generated resulting in a huge amount of available sequencing data. The data volume generated by these technologies is growing at a pace that is now challenging the storage and data processing capacities of modern computer systems [1]. The rapid improvement of sequencing technologies has enabled a number of different sequencing-based applications like genome re-sequencing, RNA-Seq, ChIP-Seq and many others [2].

The de-novo assembly of genomes and metagenomes, from fragments (i.e., reads) produced by modern sequencers, is still one of the most challenging task. Although efficient algorithms exist [3], some genomes, like plants [4], that are particularly hard to reconstruct. Usually the first step is to map the reads onto known genomes, however, if a reference genome is not available, this task cannot be carried out. For metagenomic communities the problem is even more challenging since we neither know which genomes are present in the metagenomic sample. For these reasons one of the most challenging area of research is the comparison of metagenomic microbial communities allowing derivation of important results, like the recent one by Bork et al. [5], who discovered new metagenomic markers for early-stage detection of colorectal cancer.

In the growing field of microbial communities comparison, one of the most important task is the identification of all genomes present in the mixture [7], as well as their *abundance level* [6]. Unfortunately, in these studies, we require prior information about the sample, without which identification of all genomes in the microbial community may not be possible.

In this paper we study the problem of comparing genomes and metagenomic samples directly from their reads sets without the need of costly steps like metagenomic binning, reads mappings or metagenomes assembly.

The increasing size of sequencing datasets requires ever increasing efficient algorithms. Concurrently *signature-based* methods for genome comparison (e.g., based on the frequencies of word patterns) could help analyses of genomes and metagenomes experiments from their reads data [8]. As a consequence the comparison of genomes and metagenomes, based on direct comparison of reads using alignment-free methods, has been investigated only recently [8, 9].

Alignment-free methods are receiving increasing attention because they are computationally efficient and provide attractive alternatives when alignment-based approaches fail [10]. For example the study of evolution of organisms using whole-genome sequence is impossible to conduct with traditional alignment techniques [11–13]. Some alignment-free measures use the distribution of patterns to study the identification of cis-regulatory modules (CRM) [14, 15] and of entropic profiles [16–18].

In [8] Song et al. presented the first paper on application of alignment-free techniques to the comparison of *Next Generation Sequencing* (NGS) reads sets. They showed, theoretically and with simulations, that the proposed measures, $d^{*}_{2}$ and ${d^{s}_{2}}$, are statistically more powerful than *d*_2_. In the current work we extend this study by incorporating quality values information into *d*_2_-like statistics.

Sequencing platforms produce reads, as a series of base calls and quality values. Quality values are fundamental for various analyses of reads data: mapping reads to a reference genome [19]; error correction [20]; detection of insertion and deletion [21] and many others. The new-generation sequencing technologies, like PacBio and MinION, will produce longer reads but with a larger number of erroneous bases [22], about 15 *%*. In this context the information provided by quality values will be fundamental within the alignment-free framework.

Quality values can also be used to detect and discard low quality reads for those applications that require as input sets of reads as much as possible free from errors. In the context where 15 *%* of bases could be wrong while reads become really long, it will be unlikely (if not impossible) to observe reads that are error-free. With such an increase in error rates, the ability to work with noisy reads is fundamental. Recently we proposed a set of alignment-free statistics, called *D*^*q*^-type, that are capable of comparing two reads with the intent of clustering sequencing data [23, 24]. In this work we address a different biological problem, that requires the comparison of entire sets of reads instead of just pairs of them. Since reads may come from different strands we also need to account for their reverse complements. Finally, to avoid dependencies on different length and nucleotide distributions between distribution, a dissimilarity metric must be defined.

At first we test these measures on the problem of detecting the evolutionary relationship of genomes just by comparing their sets of reads, without the need to assembly them or to have reference genomes. Then we study the ability to compare microbial communities, again, only based on metagenomic reads. For both applications we achieve promising results in terms of accuracy.

In the following section we briefly review some alignment-free measures. We start by presenting a new family of statistics for reads data, called *d*^*q*^-type, that take advantage of quality values. We then present and discuss relevant results on simulated and real data. We conclude the paper by summarizing the findings and discussing future directions of investigation.

## Previous work on alignment-free statistics

The first alignment-free method was proposed by Blaisdell in 1986 [25]. Let *X* and *Y* be two sequences from an alphabet *Σ*, for a given *k*, the value *X*_*w*_ is the number of times *w* appears in *X*, with possible overlaps. The *D*_2_ statistic is defined as: $ D_{2}= {\sum \nolimits }_{w \in \Sigma ^{k}} X_{w} Y_{w}$.

This can also be viewed as the inner product of the words vectors *X*_*w*_ and *Y*_*w*_, representing the number of occurrences of words of length *k*, in *X* and *Y* respectively. The *D*_2_ statistic can be biased by the stochastic noise in each sequence [26]. This issue has been addressed in a number of papers [14, 27, 28]. As a result several alignment-free statistics have been proposed over the years: ${D_{2}^{z}}$, $D_{2}^{*}$, ${D_{2}^{s}}$ and many others. The basic idea is to introduce a normalization factor to *D*_2_, for example ${D_{2}^{z}}$ is defined as: ${D_{2}^{z}} = \frac {D_{2} - E(D_{2})} {V(D_{2})}$, where *E*(*D*_2_) and *V*(*D*_2_) are, respectively, expectation and standard deviation of *D*_2_. However ${D_{2}^{z}}$ is still dominated by the specific variation of each pattern from the background [14, 28]. To account for different distributions of the *k*-mers, two more statistics have been introduced: $D_{2}^{*}$ [14] and ${D_{2}^{s}}$ [28]. All these measures are usually referred ti as *D*-type statistics.

Let $\tilde {X}_{w}=X_{w} - (N-k+1)*p_{w}$ and $\tilde {Y}_{w}=Y_{w} - (N-k+1)*p_{w}$ where *p*_*w*_ is the probability of word *w* under the null model and *N* is the length of *X* and *Y*. Then $D_{2}^{*}$ is defined as follows:

$$D_{2}^{*} = \sum\limits_{w \in \Sigma^{k}} \frac{\tilde{X}_{w} \tilde{Y}_{w}}{(N-k+1)p_{w}}. $$

Notice that, in the definition of $\tilde {X}_{w}$, the number of occurrences of a *k*-mer *w* is centralized by the expected number of occurrences of *w*, that is, *E*(*X*_*w*_)=(*N*−*k*+1)∗*p*_*w*_. The expectation *E*(*X*_*w*_) is an approximation under the assumption that occurrences of *w* are independent. These measures can be viewed as the standardization of the original *D*_2_. First Reinert et al. [27] and later Wan et al. [28] studied the ability of these measures to detect regulatory sequences. From the theoretical point of view, it has been shown that $D_{2}^{*}$ is statistically more powerful than *D*_2_, in the detection of relationships between sequences correlated through the common motif model where the two sequences share common motifs [27, 28].

The extension of these statistics for the comparison of NGS data has been proposed only recently [8]. The basic idea is that two genomes or metagenomes can be compared only using of their reads sets and without the need of an assembly of the reference genomes. The two major problems to be addressed are: random sampling of reads from the genomes and orientation of reads, which may come from either of the two strands of the genome. While the latter issue can be solved simply by also considering the reverse complement of a word *w*, the former is more subtle and requires a mathematical model for the sampling of reads. Both these aspects have been investigated in [8] and we report here only the final forms of the alignment-free measures *d*_2_ and $D_{2}^{*}$ for comparing of two sets of reads.

Suppose that *M* reads of length *β* are sampled from a genome. Since reads can come from either forward or reverse strand of the genome, we supplement the observed reads with their complements and refer to the joint set (i.e., reads and complement) as the reads set. Let *X*_*w*_ be the number of occurrences of the word *w* in the reads set of the genome *X* (*Y*_*w*_ is similarly defined). Recall that *X*_*w*_ accounts also for the occurrences of the reverse complements of *w*, thus, as above, one can define the average number of occurrences of *w* as $E(X_{w})=M(\beta - k+1)(p_{w}+p_{\overline {w}})$, where $\overline {w}$ is the reverse complement of *w*. Similar to the definitions of the original *D*-type statistics:

$$ D_{2}= \sum\limits_{w \in \Sigma^{k}} X_{w} Y_{w} ;\qquad D_{2}^{*} = \sum\limits _{w \in \Sigma^{k}} \frac{\tilde{X}_{w} \tilde{Y}_{w}}{M(\beta - k+1)(p_{w}+p_{\overline{w}})} $$

where $\tilde {X}_{w}=X_{w}-M(\beta - k+1)(p_{w}+p_{\overline {w}})$. These statistics cannot be directly used, as the ranges depend on several factors such as nucleotide frequencies, length and number of reads. These problems have been solved by defining the dissimilarities *d*_2_ and $D_{2}^{*}$ such that they range between 0 and 1:

$$d_{2} = \frac{1}{2} \left(\frac{D_{2}}{\sqrt{{\sum\nolimits}_{w \in \Sigma^{k}} {X_{w}^{2}} } \sqrt{{\sum\nolimits}_{w \in \Sigma^{k}} {Y_{w}^{2}} } } \right) $$

$$d_{2}^{*} = \frac{1}{2} \left(\frac{M(\beta -k+1) D_{2}^{*}}{\sqrt{{\sum\nolimits}_{w \in \Sigma^{k}} \tilde{X}_{w}^{2}/p_{w} } \sqrt{{\sum\nolimits}_{w \in \Sigma^{k}} \tilde{Y}_{w}^{2}/p_{w} } } \right). $$

These measures have been evaluated on the problem of clustering of genomic sequences and reconstruction of phylogenetic trees [8]. For simplicity in this paper we don’t consider ${D_{2}^{s}}$, since results are very similar to $D_{2}^{*}$. Also we are not interested in comparing the relative performance of these statistics, rather to understand whether or not quality values can improve the classification accuracy of all considered measures. In the next sections we lay down the basic properties of quality values and extend this theory for the comparison of sets of reads.

## Methods

### A brief introduction on quality qalues

Sequencing machines produce, for each base call of a read *x*, a *quality scoreQ*_*x*_(*i*), which represents the accuracy of that base. This score is given as *phred*-scaled probability [29] of the *i*-th base being wrong

$$Q_{x}(i) = -10 \log_{10}{Prob\{\text{the base } i \text{ of read } x \text{ is wrong } }\}. $$

For example, if *Q*_*x*_(*i*)=30 then there is 1 in 1000 chance that base *i* of read *x* is incorrect.

Similarly to the assumptions also used in [19] we will consider as independent errors at different positions of the same read. In this case the probability of the entire read *x* being correct becomes:

$$P_{x}\{\text{the read } x \text{ is correct}\}= \prod\limits_{j=0}^{\beta-1}{\left(1- 10^{- Q_{x}(j)/{10}}\right)} $$

where *β* is the length of the read *x*.

In the same way we can define the correctness probability of word *w* of length *k* occurring at position *i* of the read *x* as:

$$\begin{aligned} &P_{w,x_{i}}\{\text{the word } w \text{ at position } i \text{ of read } x \text{ is correct}\}\\ &\qquad= \prod\limits_{j=0}^{k-1}{\left(1- 10^{- Q_{x}(i+j) /{10} }\right)}. \end{aligned} $$

Alignment-free statistics based on *k*-mers frequency (like the *D*_2_-type measures discussed above) count the number of times a *k*-mer occurs in the entire sequence, regardless of the overall reliability of such occurrences. In this paper we show how quality values can be used to weight each occurrence of a *k*-mer based on the correctness probability defined above.

### New ***d***^***q***^-type statistics

In this section we extend the basic alignment-free statistics for the comparison of two sets of reads with quality values. Let *X* be the set of all reads together with their reverse complements and consider a read *x* in *X*. ${X_{w}^{q}}$ is defined as the sum of probabilities of all the occurrences of *w* in *X*; that is:

$$X_{w}^{q} = \sum\limits_{i \in \{i| w \text{ occurs in } x \text{ at position } i \text{ and }x \in X\} }P_{w,x_{i}}. $$

Thus we assign a weight (i.e., a probability) to each occurrence of *w*. The vector ${X_{w}^{q}}$ replaces the original *X*_*w*_ in the computation of all alignment-free statistics. Note that, by using ${X_{w}^{q}}$, every occurrence of *w* is not counted as 1, but with a value in [0,1] depending of the reliability of that occurrence. A new alignment-free statistics can be defined as:

$$ {D_{2}^{q}}= \sum\limits_{w \in \Sigma^{k}} {X_{w}^{q}} {Y_{w}^{q}}. $$

This can be interpreted as an extension of the *D*_2_ measure, in which two sets of reads are compared, reverse complements are considered and occurrences are weighted based on quality scores. In order to define the $D_{2}^{q*}$ statistic, we need to centralize the *k*-mers count as follows:

$$\tilde{{X_{w}^{q}}} = {X_{w}^{q}} - M(\beta -k+1)(p_{w} + p_{\overline{w}})(E(P_{w}) + E(P_{\overline{w}})). $$

If we assume that all occurrences of the word *w* are independent, the expected number of occurrences of *w* can be computed as $M(\beta -k+1)(p_{w} + p_{\overline {w}})$, where *p*_*w*_ is the probability of the word *w* and $p_{\overline {w}}$ is the probability of the reverse complement $\overline {w}$ of *w*, this same value corresponds also to the expectation of *X*_*w*_. However, in ${X_{w}^{q}}$, every occurrence of *w* does not contribute as 1, but it depends of the reliability of that occurrence. Thus we need to multiply the probability *p*_*w*_ by *E*(*P*_*w*_) which represents the expected probability of the occurrences of *w* based on the quality scores. Now that $\tilde {{X_{w}^{q}}}$ is defined, we can extend $D_{2}^{*}$ to incorporate quality values:

$$D_{2}^{q*} = \sum\limits_{w \in \Sigma^{k}} \frac{\tilde{{X_{w}^{q}}} \tilde{{Y_{w}^{q}}}}{ M(\beta-k+1)(p_{w} + p_{\overline{w}})(E(P_{w}) + E(P_{\overline{w}}))}. $$

We call these alignment-free measures *D*^*q*^-type. To estimate the expectation *E*(*P*_*w*_) we need to consider the word *w* and the distribution of quality values. The latter is highly dependent on the sequencing machine, for example quality values are not uniformly distributed, nor they follow a well defined mathematical model. Therefore it can be very hard, if not impossible, to know their distribution precisely. If the sets of reads is large enough, however, we can estimate the prior probability using the posterior relative frequency, that is, the frequency observed on the actual sets, similarly to [19]. For example we can estimate the expected quality as the average error probability of the *k*-mer *w* among all occurrences of *w* in all reads sets. Note that the value ${X_{w}^{q}}$ accounts also for the reverse complement of *w*, by definition of *X*, thus we can write:

$$E(P_{w}) + E(P_{\overline{w}}) \approx \frac{{X_{w}^{q}} + {Y_{w}^{q}}}{X_{w}+Y_{w}}. $$

More sophisticated estimators can be applied, including the redistribution of “missing” quality (see [24]) however, for simplicity, in this paper we will use only the above estimator. To avoid the dependency on different read lengths and different number of reads in the two sets (see [8]), we define the *d*^*q*^-type statistics as:

$$\begin{array}{@{}rcl@{}} d_{2}^{q} &=& \frac{1}{2} \left(\frac{{D_{2}^{q}}}{\sqrt{{\sum\nolimits}_{w \in \Sigma^{k}} {{X_{w}^{q}}}^{2}} \sqrt{{\sum\nolimits}_{w \in \Sigma^{k}} {{Y_{w}^{q}}}^{2} } } \right)\\ d_{2}^{q*} &=& \frac{1}{2} \left(\frac{M(\beta -k+1) D_{2}^{q*}}{\sqrt{{\sum\nolimits}_{w \in \Sigma^{k}} {\tilde{{X_{w}^{q}}}}^{2}/p_{w} } \sqrt{{\sum\nolimits}_{w \in \Sigma^{k}} {\tilde{{Y_{w}^{q}}}}^{2}/p_{w} } } \right). \end{array} $$

Several other alignment-free statistics can be extended with the inclusion of quality values. From the word vectors ${X_{w}^{q}}$ and ${Y_{w}^{q}}$ one can compute $d_{2}^{qs}$, the Euclidean distance *L*_2_, the Kullback-Leibler divergence *KL*, and many others. However the purpose of this paper is to evaluate improvements obtained by using quality values with respect to the traditional *d*-type measures. All measures can be computed in linear time and space, which is desirable for large NGS datasets. The software *c*2*q* (http://www.dei.unipd.it/~ciompin/main/c2q.html), that computes both *d*_2_ and ${d_{2}^{q}}$ type statistics, will be briefly described at the end of experimental results section.

## Results and discussion

Alignment-free measures have already been proved effective in solving many biological problems. One of such problems, which has been extensively investigated, is the reconstruction of phylogeny tree based on the comparison of reads data [8, 9, 30]. Such application leverages the power of alignment-free statistics on clustering [23, 24, 31] biological sequences (e.g., reads) which makes them suitable for metagenomic data where one of the most important task requires partitioning (i.e., clustering) data based on their genome. The problem of labeling reads produced by metagenomic experiment with information about their origin genome (*metagenomic reads binning*), can not be solved if the genomes populating the sample are unknown. Current state of the art methods require first a (possibly draft) assembly of all genomes and then reads are aligned against assembled contigs [6]. In this scenario alignment-free measures, which can be computed in linear time on the total number of bases, represent a valuable alternative to directly compare two or more metagenomes without the need of costly alignments or assemblies. For these reasons we tested the *d*_2_-type measures presented in the previous sections as tools to perform phylogeny tree reconstruction and metagenomic comparison based only on reads data.

### Reconstructing evolutionary relationships from reads data

We present here results for *d*_2_-type statistics when applied to the problem of phylogeny tree reconstruction, which has recently received increasing interest thanks to the development of several alignment-free statistics [8, 9, 30] and to the availability of excellent tools (e.g., rose and PHYLIP described below) for generation of simulated data and analysis of phylogeny trees. Using these tools in combination with our c2q software, we developed an experimental pipeline divided into three phases: *sequences generation*, *reads sampling* and *distance calculation*; these phases are briefly described below; the entire pipeline is similar to the one used in [30].

We start with the *sequences simulation* phase which uses rose software [32] to generate families of sequences that are organized in a phylogeny tree with a *root sequence* as common evolutionary ancestor. This tree is also outputted by the software and is used in latter phases for comparison with the reconstructed trees. We set rose so to generate a total of 50 sequences with all parameters at their default values except for the *relatedness* (which is the average evolutionary distance between sequences) that has been set to 70 *Point Accepted Mutations (PAM)* units, relatedness is used by rose to construct the mutation matrix.

The second phase of the pipeline, the *reads sampling*, is performed for each sequence generated in the previous step. This phase has been performed using mason reads simulator [33] with *illumina* preset and all parameters set to their default values except for the *mismatch probability* (parameter pmm) that has been set to 0.1 (i.e., 10 % mutation rate). Such a high error rate has been chosen to emphasize the presence of errors (against which our ${d_{2}^{q}}$-type measures should be robust) with the idea that these measures may be used with future generation sequencing (like PacBio and MinION) that produce long and noisy reads. Among the many possible reads simulators we chose mason because it is one of the few softwares able to produce quality scores (essential to compute our measures), while still based on empirical profiling of reads. After reads have been simulated, the 50 sets produced by mason (one for each sequence) are used as input to our c2q software that computes dissimilarity matrices of the four statistics *d*_2_, $D_{2}^{*}$, ${d_{2}^{q}}$ and $D_{2}^{q*}$ presented in the previous sections. These 50×50 matrices are used to construct a phylogeny tree with neighbor software from the PHYLIP package [34]. Such software allows the matrix to be constructed using either *Unweighted Pair Group Method With Arithmetic Mean (UPGMA)* or *Neighbor Joining (NJ)*. In this paper we present only results on UPGMA which, in our setup, exhibits more stable behavior as the parameters vary, however results for NJ are very similar and same conclusions could be drawn from them. The last step of *distance calculation* phase takes all the four trees along with the original one (produced by rose) and computes the *Robinson-Foulds symmetric distance* (from now on R-F) between all the pairs of trees by using the treedist software of the PHYLYP package. The R-F distance is equal to 0 if the trees are isomorphic, and it increases if the trees a different. To make our results less dependent from the initial random generation of the root sequences, the entire pipeline is executed 10 times and the average R-F distance is here presented.

Performance of *d*_2_ measures must be tested under two aspects. The first is the variability of input data characteristics in terms of sequence length *N*, number of reads *M* and length of reads *β*, in some cases it is useful to refer to the coverage defined as *βM*/*N*. The second aspect is the sensibility of results with respect to the parameter *k*.

**Fig. 1:**
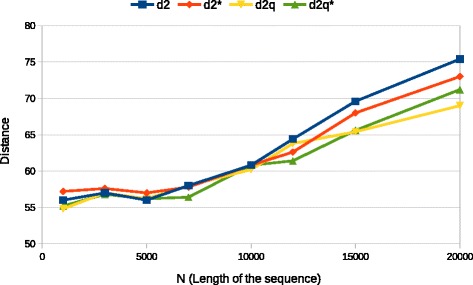
Average R-F distance between estimated and original trees as sequence length *N* varies

We start by testing robustness of the statistics as the parameters of input data change. The first experiment varies the length *N* of the sequences, results are presented in Fig. 1 (in this set of experiments *β*=300, *M*=300 and *k*=7). The considered values of *N* are between 1000 and 20000 which is roughly the same range used in [8, 30]. As expected the performance of measures in terms of the average R-F distance becomes worse as *N* increases, there are two phenomena involved in this behavior: the first is the decrease in coverage induced by *N* and the second is the presence of more *k*-mers in the background sequence. We see, however, how the $D_{2}^{*}$ measures (with and without quality) are more robust against the increase of *N*, moreover, in general, $D_{2}^{q*}$ performs better than all other measures suggesting that for lower coverage this measure is the best choice. For all subsequent experiments we keep fixed *N*=5000 as this appear to be the optimal value for the setup we considered.

**Fig. 2:**
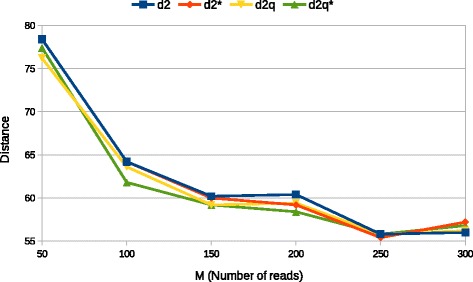
Average R-F distance between estimated tree and the original as number of reads *M* varies

We next present results when the number of reads *M* varies, Fig. 2 shows how the average R-F distance varies with *M* (in this test case *β*=300, *N*=5000 and *k*=7). The trend we observe is similar to one with variable *N* suggesting that *d*_2_ measures are more sensible to overall coverage rather than to the variation of single parameters. The sharp improvement from *M*=50 to *M*=100 is likely due to the fact that in the former the coverage is 1 therefore no redundancy is present in the input data. Again the figure shows that $D_{2}^{q*}$ performs slightly better than the other measures. Although both values 250 and 300 seems to give optimal performance, to privilege higher coverage, we chose the latter as base value for all other experiments.

**Fig. 3:**
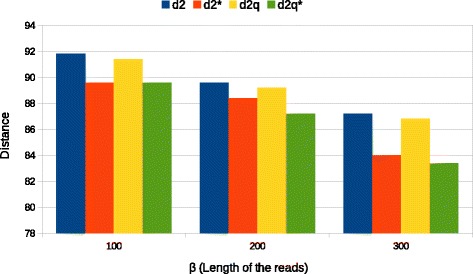
Average R-F distance between estimated tree and the original as the length of reads *β* varies

In Fig. 3 is shown the impact of reads length on the average R-F distance between reconstructed and real trees (*k*=5, *N*=10000 and *M*=200). This set of experiments again confirms that higher coverage (in this case obtained increasing the length of reads) gives better results. In general, however, the increase in terms of absolute R-F distances is not as evident as the one observed when increasing the number of reads *M*. This can, partly, be explained by the fact that, for *k*≪*β*, the length of reads gives marginal benefits and, for fixed coverage, one may prefer to sequence a larger amount of shorter reads. Also these experiments confirm that the relative performance between different *d*_2_ statistics is maintained as in previous experiments. The choice of a different type of chart in Fig. 3 has been made to overcome the smaller scale of R-F distance which may compromise the overall readability.

**Fig. 4:**
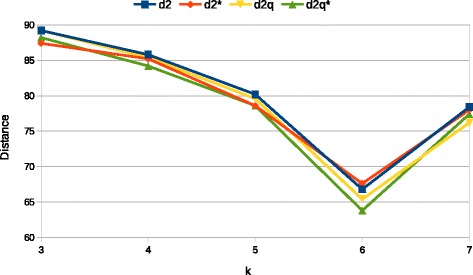
Average R-F distance between estimated and original trees as parameter *k* varies

After having discussed performance of *d*_2_ measures as the data parameters vary, we consider now the sensibility to the only parameter *k* that characterizes these measures. Figure 4 shows the results we obtained for values of *k* between 3 and 7, which are the same values that have also been tested in [8] (other parameters are set to *N*=5000, *M*=300 and *β*=300). We observe a general improvement while *k* increases with the statistics based on quality values performing slightly better. The minimum, observed for *k*=6, is compatible with conclusions drawn in [8] where, in a similar setup, optimal values of *k* are found to be between 4 and 6. A possible explanation of this optimal *k* could be based on the length of sequence *N*=5000 for which, by assuming a uniform model, substrings of length less than 6 are expected to appear more than once on average while strings of length greater than 7 occurs on average a number of times less than one (the real threshold for which strings would be expected to appear exactly one is log45000≈6.14). This may indicate that *d*_2_ measures give good results when the expected number of times a *k*-mer appears is around one, this observation, however, needs more evidence to be a complete explanation of the minimum observed in Fig. 4.

### Fast classification of metagenomes

Next we consider the application of *d*_2_ measures to the comparison of reads sets coming from different metagenomic experiments. We want to test if “similarity” between two metagenomes can be detected without knowing the genomes in the mixture. Complete binning of all reads can be very demanding, requiring several GB of memory and CPU hours [35]. We show here how fast comparison of metagenomes is still possible by using alignment-free measures.

To this extent we generated 6 different types of metagenomes, based on real genomes, using SynMetaP (https://bitbucket.org/CibioCM/synmetap/). The 6 types of metagenomes are paired into three groups, each group has been created to test a specific characteristic of metagenomic data. More precisely each type of metagenomes contains 10 genomes and groups are created based on the percentage of shared organisms and on different abundance profiles. For each type of metagenomes 5 different metagenomes are sampled, so that for every experiments we have 10 metagenomes to compare, 5 from each type. In the first experiment we compare two types of metagenomes, one containing 10 genomes from *Lactobacillus* bacteria and the other 10 genomes from *Vibrionaceae* bacteria, all with the same abundance rate. This group (called *LV*) represents the ideal situation where two distinct communities of organisms are sequenced within the same experiment. This scenario should be effectively resolved by *d*_2_ measures because different species have significant different *k*-mers count statistics. The second experiment (called *M*) compares two types of metagenomes, each of 10 genomes, that share 5 genomes, all the genome are present with the same abundance. Finally in the last setup (called *MA*) the two types of metagenomes contains the same 10 genomes but with different abundance profiles, this is the more difficult and realistic test. For the last two groups of metagenomes, *M* and *MA*, we used the same datasets used to test Kraken software [35] (MiSeq and HiSeq metagenome, see Table S1 therein), one of the best method for metagenomic reads binning.

In this section we present data only for $D_{2}^{q*}$ since it provides the best result and all the other measures expose similar behavior while maintaining performance relative to each other compatible with what described in previous sections.

**Fig. 5:**
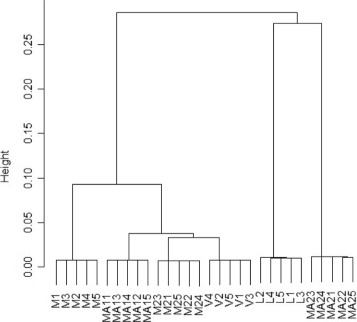
Output dendogram of hierarchical clustering when dissimilarity matrix is produced using $D_{2}^{q*}$ measure (*k*=8) on all the datasets for the three groups *LV*, *M* and *MA*

For each experiment, in order to evaluate how well we can distinguish similar metagenomes, we perform hierarchical clustering based on the dissimilarity measure produced by $D_{2}^{q*}$. For every test a tree like structure, called *dendogram* (see Fig. 5), is constructed, where each leaf represents a metagenomes. Branches of the dendogram are weighted by the dissimilarity between the two associated subtrees, such weight can be interpreted as height of internal nodes and the nodes of a dendogram are plotted accordingly. Upon a correct classification, the higher the node that defines the correct clustering, the better the measure discriminates.

The choice of dendogram for visualizing performance of the algorithm, has been made because the accuracy of reconstructed trees is 100 *%*, that is, each metagonme were correctly assigned to the corresponding cluster. Other types of graph (for example ROC) would have been meaningless as the true positive rate is always 100 *%* for all our simulations.

**Fig. 6:**
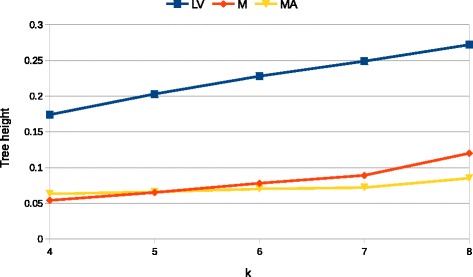
Height of the node that correctly clusters the two types of metagenomes for the groups *LV*, *M* and *MA* as a function of parameter *k* of the $D_{2}^{q*}$ measure used to produce the dissimilarity matrix

We tested the $D_{2}^{q*}$ measure on the above described metagenomic sets by measuring the height of the node that achieves the ideal partition (i.e., the correct one). Figure 6 shows these heights for the three groups described above. As expected the *LV* group, being easier to classify, attains significantly higher values compared to the other two groups. For *M* and *MA* we observe very similar results suggesting that $D_{2}^{q*}$ measure has low sensitivity to the abundance profile. From Fig. 6 we also see that the height of the discriminating node increases with parameter *k*, this confirms that, by considering longer *k*-mers, the discriminating power of $D_{2}^{q*}$ (as all *d*_2_) measure increases. The magnitude of such increase, however, is highly variable, while on group *M* by doubling *k* (from 4 to 8) the height more than doubles (from 0.054 to 0.121), on group *MA* the same change in *k* produces roughly a 40 *%* increase (from 0.063 to 0.085). In summary in all three experiments we are able to correctly cluster similar metagenomes, but with different accuracies depending on the difficulty of the datasets.

In the previous experiments we tested how well we can discriminate two different types of metagenomic samples. Another interesting application is the comparison of all 6 different types of metagenomes, from which we have a total of 30 metagenomic samples. Thus we performed a further test where all the samples of all types are mixed together and compared through clustering. The resulting tree, constructed from the $D_{2}^{q*}$ dissimilarity matrix for *k*=8, is shown in Fig. 6. Surprisingly all the samples from the same metagenomic type are clustered together, thus confirming that similar metagenomes can be deteceted even if they are mixed in a relatively big dataset. Moreover this reconstruction is fairly consistent with the expected one, in which the groups *M* and *MA*, that shares some genomes, should be closer. The only notable exception is the group *MA*2 that is incorrectly classified distant from sets *MA*1.

### Our c2q software

Our c2q software has been developed starting from the c2 software used in [8] by adding support to quality value based measures ${d_{2}^{q}}$ and $D_{2}^{q*}$. The software takes *n*fastq files as input (listed in a proper text file) and produces several matrices as output. More precisely a total of eight *n*×*n* symmetric matrices are computed by the software; four for all the *D*_2_ measures and four for the *d*_2_ measures (the former are used to compute the latter and are outputted for convenience). These matrices can be used to classify the *n* reads sets, in our case they have been inputted to PHYLIP to reconstruct phylogeny trees and to a hierarchical clustering algorithm.

The software scans input files one at the time and counts frequency of *k*-mers with and without weighting them based on quality values (see sections above) and therefore the overall time complexity is *O*(*βM*). For time efficiency purpose, original software c2 requires space proportional to 4^*k*^, for this reason we were not able to test measures on large values of *k*, however for small *k* the software can be run on commodity hardware (e.g., laptop) which is not the usual case for metagenomic software that always require considerable amount of resources. For example kraken [35] software is based on the construction of a database that requires more than 70 GB of main memory and takes many CPU hours to be built.

We believe that the availability of fast tools for metagenomic reads classification will help interpreting data to obtain rough indication of the genomes present in the communities and of their characteristics in a more timely way. Our c2q software (especially for small *k*) can be used for this first coarse classification step and could be applied as pre-filtering step to other more compute intensive phases if more fine results are needed. As an indication of performance, we measured execution time between 92 and 94 seconds when constructing 50×50 matrices for the first sets of experiment (i.e., phylogeny tree reconstruction) with *k*=7 (the highest we tested) on a Linux desktop machine equipped with a quad-core 3.1 GHz AMD processor and 4 GB main memory. The software is written in C++ and has been compiled with gcc 4.7.2 with -O3 optimization flag, no particular libraries are required and the source code is portable to any platform.

## Conclusions

The comparison of sets of reads using quality values is essential in many genomic projects. When reference sequences are not available, or difficult to assemble, comparison of reads sets can be used to detect evolutionary relationships between the underlying genomes and metagenomes. We believe that, with the advent of future sequencing technologies, where the error rates are about 15 %, the importance of quality values will considerably increase. In this work we presented a family of alignment-free measures, called *d*^*q*^-type, that incorporate quality values information in *k*-mers count statistics and that can be used for the comparison of sets of read data. Experiments on simulated genomes show that these statistics can be used to detect the evolutionary relationships among genomes just by comparing their reads. The use of quality values within alignment-free measures on average improves the classification accuracy and the impact of quality values increases when the reads are more noisy and the coverage is low.

Preliminary experiments on metagenomic reads data show that also similar metagenomes can be correctly classified, without even knowing the genomes contained in each sample. Although a more comprehensive evaluation on large metagenomes can be of interest, this seems to be a promising area of investigation, where alignment-free techniques can be used as a rough filter to cluster together similar metagenomes.

As future work we plan to further explore the application of alignment-free statistics in the context of metagenomes, in particular for the problems of genome diversity estimation and binning.

## References

[1] Medini D, Serruto D, Parkhill J, Relman D, Donati C, Moxon R (2008). Microbiology in the post-genomic era. Nat Rev Microbiol.

[2] Jothi R, Cuddapah S, Barski A, Cui K, Zhao K (2008). Genome-wide identification of in vivo protein-DNA binding sites from ChIP-Seq data. Nucleic Acids Res.

[3] Zerbino DR, Birney E (2008). Velvet: algorithms for de novo short read assembly using de Bruijn graphs. Genome Res.

[4] Schatz MC, Witkowski J, McCombie WR (2012). Current challenges in de novo plant genome sequencing and assembly. Genome Biol.

[5] Zeller G, Tap J, Voigt A, Sunagawa S, Kultima J, Costea P (2014). Potential of fecal microbiota for early-stage detection of colorectal cancer. Mol Syst Biol.

[6] Wang Y, Leung HC, Yiu SM, Chin FY (2014). MetaCluster-TA: taxonomic annotation for metagenomic data based on assembly-assisted binning. BMC Genomics.

[7] Segata N, Börnigen D, Morgan XC, Huttenhower C (2013). PhyloPhlAn is a new method for improved phylogenetic and taxonomic placement of microbes. Nat Commun.

[8] Song K, Ren J, Zhai Z, Liu X, Deng M, Sun F (2013). Alignment-free sequence comparison based on next-generation sequencing reads. J Comput Biol.

[9] Comin M, Schimd M (2014). Assembly-free genome comparison based on next-generation sequencing reads and variable length patterns. BMC Bioinformatics.

[10] Vinga S, Almeida J (2001). Alignment-free sequence comparison – a review. Bioinformatics.

[11] Gregory ES, Se-Ran J, Guohong AW, Sung-Hou K (2009). Alignment-free genome comparison with feature frequency profiles (FFP) and optimal resolutions. PNAS.

[12] Comin M, Verzotto D (2012). Whole-genome phylogeny by virtue of unic subwords. Proc. 23rd Int. Workshop on Database and Expert Systems Applications (DEXA-BIOKDD’12).

[13] Comin M, Verzotto D (2012). Alignment-free phylogeny of whole genomes using underlying subwords. BMC Algorithms Mol Biol.

[14] Kantorovitz MR, Robinson GE, Sinha S (2007). A statistical method for alignment-free comparison of regulatory sequences. Bioinformatics.

[15] Comin M, Verzotto S (2014). Beyond fixed-resolution alignment-free measures for mammalian enhancers sequence comparison. Proc Twelfth Asia Pacific Bioinformatics Conference IEEE/ACM Trans Comput Biol Bioinformatics.

[16] Comin C, Antonello M (2013). Fast computation of entropic profiles for the detection of conservation in Genomes. Proc Pattern Recognit Bioinformatics PRIB Lecture Notes in Bioinformatics.

[17] Comin M, Antonello M (2014). Fast Entropic Profiler: An information theoretic approach for the discovery of patterns in Genomes. IEEE/ACM Trans Comput Biol Bioinformatics.

[18] Comin M, Antonello M. Fast alignment-free comparison for regulatory sequencesusing multiple resolution entropic profiles. Proc Int Conf Bioinformatics Models Methods Algorithms. 2015:171–7.

[19] Heng L, Jue R, Durbin R (2008). Mapping short DNA sequencing reads and calling variants using mapping quality scores. Genome Res.

[20] Hashimoto WS, Morishita S (2009). Efficient frequency-based de novo short-read clustering for error trimming in next-generation sequencing. Genome Res.

[21] Albers C, Lunter G, MacArthur DG, McVean G, Ouwehand WH, Durbin R (2011). Dindel: accurate indel calls from short-read data. Genome Res.

[22] Carneiro MO, Russ C, Ross MG, Gabriel SB, Nusbaum C, DePristo MA (2012). Pacific biosciences sequencing technology for genotyping and variation discovery in human data. BMC Genomics.

[23] Comin M, Leoni A, Schimd M (2014). QCluster: Extending Alignment-Free Measures with Quality Values for Reads Clustering. Proc WABI 2014 Lecture Notes Comput Sci.

[24] Comin M, Leoni A, Schimd M (2015). Clustering of reads with alignment-free measures and quality values. BMC Algorithms Mol Biol.

[25] Blaisdell BE (1986). A measure of the similarity of sets of sequences not requiring sequence alignment. PNAS.

[26] Lippert RA, Huang HY, Waterman MS (2002). Distributional regimes for the number of k-word matches between two random sequences. PNAS.

[27] Reinert G, Chew D, Sun F, Waterman MS (2009). Alignment-free sequence comparison (I): statistics and power. J Comput Biol.

[28] Wan L, Reinert G, Chew D, Sun F, Waterman MS (2010). Alignment-free sequence comparison (II): theoretical power of comparison statistics. J Comput Biol.

[29] Ewing B (1998). Green, E. Genome Res.

[30] Leimeister C, Morgenstern B (2014). kmacs: the k-mismatch average common substring approach to alignment-free sequence comparison. Bioinformatics.

[31] Solovyov A, Lipkin WI (2013). Centroid based clustering of high throughput sequencing reads based on n-mer counts. BMC Bioinformatics.

[32] Stoye J, Evers D, Meyer F (1998). Rose: generating sequence families. Bioinformatics.

[33] Holtgrewe M. Mason–a read simulator for second generation sequencing data. Technical Report FU Berlin 2010. http://publications.mi.fu-berlin.de/962/.

[34] Felsenstein J (1989). Phylip-phylogeny inference package (version 3.2). Cladistics.

[35] Wood DE, Salzberg SL (2014). Kraken: ultrafast metagenomic sequence classification using exact alignments. Genome Biol.

